# Refinement of the Central Steps of Substrate Transport by the Aspartate Transporter GltPh: Elucidating the Role of the Na2 Sodium Binding Site

**DOI:** 10.1371/journal.pcbi.1004551

**Published:** 2015-10-20

**Authors:** SanthoshKannan Venkatesan, Kusumika Saha, Azmat Sohail, Walter Sandtner, Michael Freissmuth, Gerhard F. Ecker, Harald H. Sitte, Thomas Stockner

**Affiliations:** 1 Institute of Pharmacology, Center for Physiology and Pharmacology, Medical University of Vienna, Vienna, Austria; 2 Division of Drug Design & Medicinal Chemistry, Department of Pharmaceutical Chemistry, University of Vienna, Vienna, Austria; Pierre and Marie Curie University (UPMC), FRANCE

## Abstract

Glutamate homeostasis in the brain is maintained by glutamate transporter mediated accumulation. Impaired transport is associated with several neurological disorders, including stroke and amyotrophic lateral sclerosis. Crystal structures of the homolog transporter GltPh from *Pyrococcus horikoshii* revealed large structural changes. Substrate uptake at the atomic level and the mechanism of ion gradient conversion into directional transport remained enigmatic. We observed in repeated simulations that two local structural changes regulated transport. The first change led to formation of the transient Na2 sodium binding site, triggered by side chain rotation of T308. The second change destabilized cytoplasmic ionic interactions. We found that sodium binding to the transiently formed Na2 site energized substrate uptake through reshaping of the energy hypersurface. Uptake experiments in reconstituted proteoliposomes confirmed the proposed mechanism. We reproduced the results in the human glutamate transporter EAAT3 indicating a conserved mechanics from archaea to humans.

## Author Summary

We used the archaeal homolog GltPh of the human glutamate transporters to refine our understanding how large scale conformational changes are translated into substrate translocation. We identified the structural changes that accompany substrate transport and convert the energy stored in the ion gradient into a directional transport. Insights into the mechanics of these transporters are likely to increase our understanding of how they contribute to excitotoxicity and to develop drugs, which preclude the underlying accumulation of glutamate in the synaptic cleft.

## Introduction

Glutamate is the primary excitatory neurotransmitter in the central nervous system. Excitatory amino acid transporters (EAAT) are membrane proteins, which remove released glutamate from the synaptic cleft [1]. Substrate transport by the human EAATs is coupled to transport of three sodium ions and one proton followed by the counter-transport of one potassium ion [2–4]. Persistent elevation of extracellular glutamate can lead to excitotoxicity through neuronal cell death. Dysfunction in EAATs has been implicated in several neurological diseases, which are associated with loss of neurons (*e*.*g*., amyotrophic lateral sclerosis, Alzheimer’s disease, stroke, cerebral ischemia, traumatic brain injury, epilepsy and Huntington’s disease) [5–9]. Development of drugs, which could enhance EAAT transporter activity, would be beneficial for patients.

The aspartate transporter from *Pyrococcus horikoshii* (GltPh) [10–13] is a homolog of the mammalian EAATs. GltPh and human EAATs share ~36% sequence identity. Sequence conservation is higher for residues implicated in substrate and ion binding [14–18]. The first crystal structure of the trimeric GltPh was solved in the outward-occluded state [10], followed by structures determined in three additional conformations [11–13,19]: outward-open, inward-occluded and intermediate, revealing a large scale translational motion during substrate transport [12]. The transport domain contains substrate and ion binding sites, while the trimerization domain mediates the contact between the protomers. Functional studies indicated that transport of one substrate molecule is coupled to the co-transport of three sodium ions [20,21]. The respective sodium binding sites were termed Na1, Na2 and Na3.

Simulations of the outward-occluded and the inward-occluded conformation have been used to study binding of substrate and co-transported ions [22–24]. The importance of the HP1 and HP2 gates for restricting the accessibility to the substrate-binding site has been studied using molecular dynamics (MD) simulation [23,25–28]. The Na3 sodium-binding site [23] has been predicted by MD simulations and was experimentally validated in GltPh and EAATs [24,29–31]. The conformational changes of substrate translocation have been studied by motion planning, elastic network model and metadynamics [32–34].

A comparision of the outward and inward facing crystal structures (PDB ID: 2NWX, and 3KBC) suggested a nearly rigid body movement of the transport domain vs. the trimerization domain. We used steered molecular dynamics (SMD) simulations to investigate the substrate translocation step from the outward-facing to the inward-facing conformation. We identified local events that are essential for coupling of the ion gradient to substrate transport and verified our observations by experiments. Repeated SMD simulations of 0.3 μs length revealed the existence of an intermediate state, which is characterized by maturation of the Na2 binding site. Maturation required changes in the local environment, which are triggered by rotation of the side chain of T308. We provide biochemical evidence for the localization and the functional role of the Na2 site: (i) mutation of residue T308 increased K_m_ for substrate and sodium in GltPh and in human EAAT3. (ii) The Hill coefficient of sodium dependence was reduced from 2.5 for wild type to 1.7 for T308V and 1.1 for T308A confirming the location of the Na2 site and its role in coupling of the sodium gradient to substrate uptake.

## Results

We inserted the outward-occluded (PDB ID: 2NWX) [11] and the inward-occluded (PDB ID: 3KBC) [12] GltPh crystal structures (both apo and in complex with substrate and sodium ions) into pre-equilibrated membranes. The conformations remained stable in two independent 100 ns long simulations. The root mean square deviation (RMSD) of the TM helices (Cα atoms) reached 0.08–0.12 nm per protomer. Metastable states (in the time window of the simulations) were expected, because the transport rate of GltPh [35] is in the range of 0.3 min^-1^. Simulations of the outward-occluded state revealed that the HP2 loop opened in the absence of substrate and ions. The observed gate opening is consistent with experimental data, which showed that closing of the HP2 loop requires binding of substrate and sodium ions[23,25,26,36].

Substrate and the sodium ions bound to the Na1 and Na3 sites remained close to their respective initial positions. In contrast, the sodium ion bound to the Na2 site could not be maintained in its position and diffused into the solvent during equilibration. Dissociation of sodium from the Na2 site occurred in all protomers in every simulation of the outward-occluded state (also in the SMD simulations described below). In contrast, it remained bound in the simulations of the inward-occluded state. Several modifications were applied to the release protocol, but we did not obtain a sodium ion stably bound to the crystallographically observed Na2 site. Similar observations were reported by others [22,37]. A Na2 site that was shifted by ~0.3 nm relative to the position of the crystallographically observed Tl^+^ ion was recently proposed [22].

### Global movement of domains during transport

**Fig 1:**
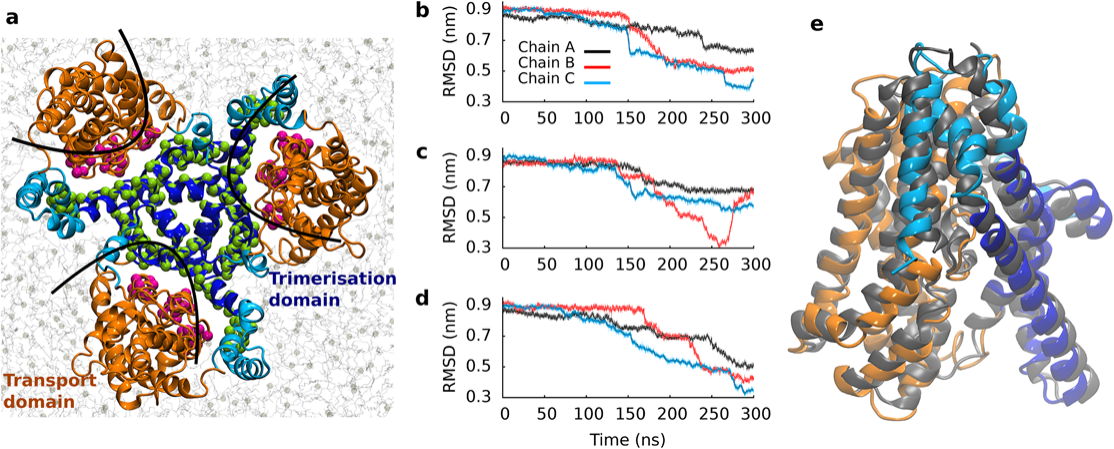
Global structure of GltPh. (a) Membrane embedded trimeric transporter structure, seen from the extracellular site. The transport domain is shown in orange, the trimerization domain in light (membrane exposed) and dark blue (without membrane contacts). The same color code is applied to all figures. The backbone atoms used as pulling groups are highlighted in green and purple. (b-d) Time evolution of transporter geometry is shown of three independent simulations, measuring the structural deviation of each protomer individually (chain A in black, chain B in red, chain C in blue) from the inward-facing crystal structure. (e) Overlay of the inward-facing crystal structure in gray (PDB ID: 3KBC) and the closest conformation extracted from the SMD simulation. The RMSD between the structures was 0.35 nm.

Substrate uptake by GltPh involves a large conformational change [12], which exposes the substrate binding site alternatingly to the extracellular or intracellular side of the membrane, consistent with the alternating access models [38]. The equilibrium simulations indicated that the transport process is too slow to be investigated directly. We therefore used SMD simulations to accelerate the transition. Each SMD simulation was repeated 3 times. The crystal structures (PDB ID: 2NWX and 3KBC) provided the framework for defining the reaction path [11–13]. They show that transport and trimerization domain undergo a translational motion relative to each other. The reaction path is almost perpendicular with respect to the membrane. We therefore applied a force on both domains in opposing directions, oriented perpendicular to the membrane plane. The force was applied to the backbone atoms of residues in the region that forms the domain interface, selecting residues K266-V274, L282-K290, I298-L305 of the transport domain and residues L54-G69, P153-Y167, K178-A214 of the trimerization domain (Fig 1A). Selection of the interface region was motivated by the minimal torque induced in case of side chain entanglements at the domain interface. Trial simulations with several different pull group selections supported this choice. The pulling velocity applied was set to 0.01 pm ps^-1^. Faster pulling rates with the same and with stronger spring constants were also tested, but resulted in increasingly unstable behavior (S1 Fig). Our simulations are well behaved, as evident from the force vs. time and distance vs. time plots (S2 Fig). We monitored the time evolution of each protomer (chain) by measuring the RMSD with respect to the inward-occluded state as observed in the crystal structure (PDB ID: 3KBC) [12] (Fig 1B, 1C and 1D). The RMSD between the outward-occluded and the inward-occluded state is 0.90 nm. A decrease to 0.3–0.5 nm indicated a successful transition to the inward-occluded state with essentially overlapping secondary structure elements (Fig 1E). The RMSD between the inward facing crystal structure (PDB ID: 3KBC) and the overlaid MD snapshot was 0.35 nm. Structure and conformation of the individual domains remained close to the initial conformation (S3 Fig), indicating that the structure of the domains remained intact. The final rise of the RMSD towards the end of the simulations, after reaching the inward-occluded state, reflects pulling of the transporter beyond the inward-facing state, while reaching the 300 ns defined as simulation length. It marks the transition from conformational changes as part of the transport cycle to distortions (S3 Fig) induced by the SMD protocol. The caesura observed in the structural stability of the individual domains by pulling beyond the inward-facing state also indicates that we were able to move the transporter along the low energy path for the investigated transition from the outward-facing to the inward-facing state.

The inward-occluded state was crystallized with the help of a conformation stabilizing double cysteine mutant (K55C and A364C). We observed a transition to the inward-facing state of at least one protomer in each simulation. Thereby residues K55 and A364 were found to come into close proximity (See S4 Fig). A second chain reached the inward-facing state in two instances. The third chain showed only once a transition to the inward facing state. The extracellular gate of the A chain is more open in the crystal structure (PDB ID: 2NWX) (See also S3A–S3C Fig). For simplicity, below we discuss results of only one simulation. Analyses of the other two simulations lead to the same conclusions (S5 and S6 Figs).

**Fig 2:**
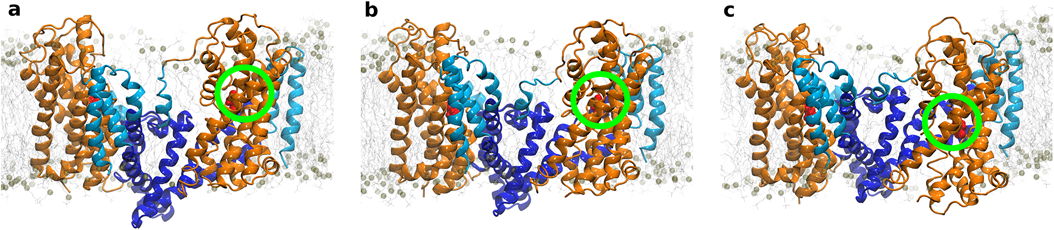
Conformation of GltPh during substrate translocation. Snapshots of the transporter in (a) the outward (at 0 ns), (b) the intermediate (at 152 ns) and (c) the inward-facing state (at 272 ns): the transport domain remained in contact with the membrane and translated minimally relative to the membrane normal, while the trimerzation domain moved towards the cell exterior.

We observed a progression towards the inward-occluded state in two global steps. Representative snapshots (Fig 2A, 2B and 2C) are depicted at three time-points, representing the outward-occluded (at 0 ns), the intermediate (at 152 ns) and the inward-occluded state (at 272 ns). The structural overlap with the crystallized intermediate structure (PDB ID: 3V8G) was largest in the intermediate conformation of our trajectories (S7 Fig). The observed structural rearrangements showed a movement of the transport domain (orange) relative to the trimerization domain (blue). The transport domain is in direct contact with the membrane; it moved as a rigid body (S3 Fig), and translated only minimally relative to the membrane. In contrast, the core of GltPh (TM2, TM4 and TM5), which showed minimal contacts with the membrane, displayed a large movement towards the extracellular side. This was accompanied by a switch in accessibility of the substrate binding site from extracellular to intracellular. We identified two distinct dynamic subdomains within the trimerization domain. A comparison between available crystal structures (PDB ID: 2NWX, 3V8G and 3KBC) suggested that the trimerization domain could bend within helices TM2 and TM5. We observed that the central part of the trimerization domain moved relative to the transport domain. In contrast, the membrane-exposed helices TM1 and the membrane adjacent parts of TM2 and TM5 did not translocate. Instead, the trimerization domain bent at residues P45-G47, P60 in TM2 and P206-G208 in TM5 to allow the membrane-exposed part to maintain membrane contacts.

### The Na2 site matured during substrate transport

**Fig 3:**
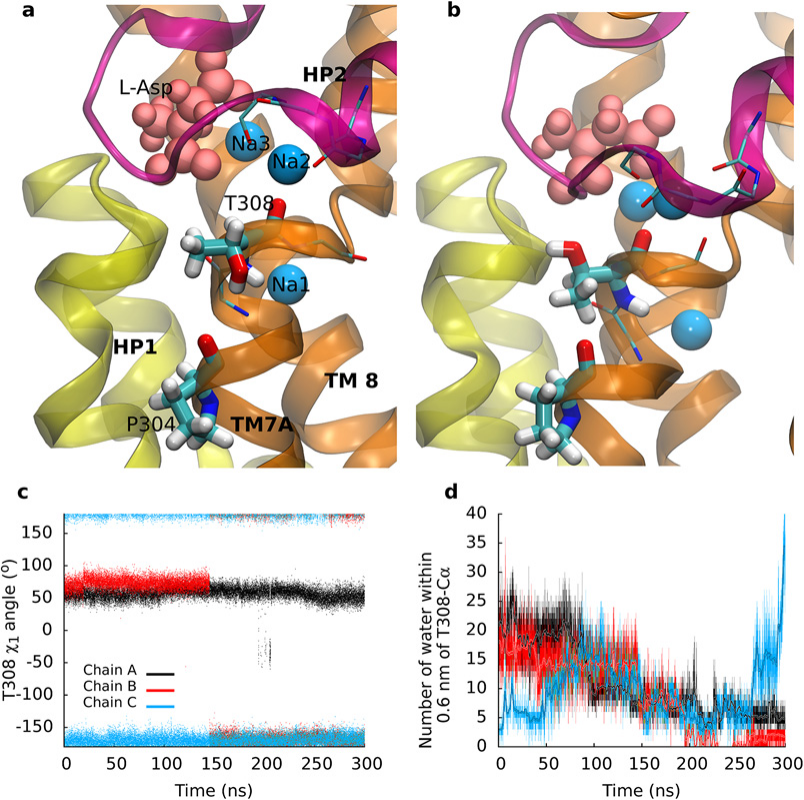
The Na2 site. (a) Comparison of the Na2 site in the outward-facing conformation before and (b) after rotation of T308. The HP1 re-entrance loop is highlighted in yellow, the HP2 loop in purple. Local changes allowed for an optimal coordination of the bound sodium. (c) Time evolution of the side chain dihedral angle of T308. (d) Level of hydration of T308 measured by the number of water molecules within 0.6 nm from the Cα atom.

We observed that sodium bound to the Na2 site was unstable in the crystallographically observed position of the outward-occluded state (Fig 3A). The side chain of T308 rotated by 120 degrees in the course of the SMD simulations (Fig 3B). The same re-rotation did not occur in equilibrium simulations. Residue T308 is adjacent to the crystallographically observed Na2 site and located on the last turn of the helix TM7A. The contiguous NMDG motif was implicated in substrate and ion binding [15,39]. The hydroxyl group of the T308 side chain formed a hydrogen bond with the backbone carbonyl of P304 on the preceding helical turn. Breaking of the P304-T308 hydrogen bond triggered rotation of the T308 side chain (Fig 3C). The transition correlated with a reduced hydration of T308 (Fig 3D).

**Fig 4:**
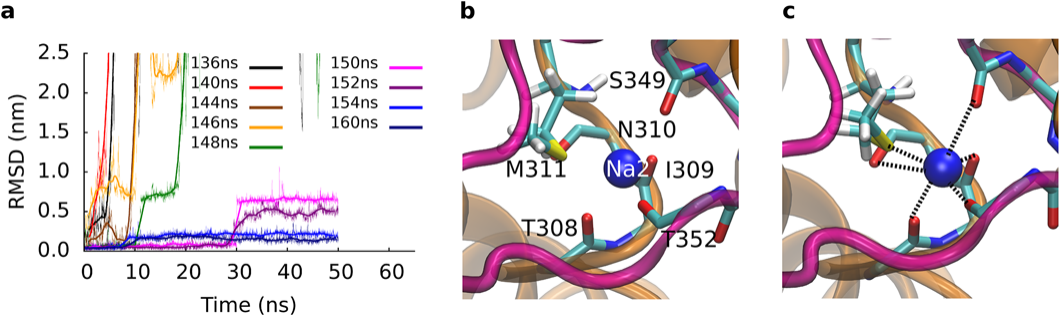
Stabilizing sodium to the Na2 site. We extracted closely spaces snapshots before and after the rotation of T308 and carried out 50 ns long simulations. (a) The RMSD of sodium 2 reflects the stability of the reinserted sodium. The time evolution of the RMSD can be classified into two groups: Large RMSD and unbinding before T308 rotation, and sodium bound to the Na2 site after rotation of T308. Panel (b) and (c) show the initial and final structure of the simulation initiated for the snapshot extracted at 160 ns. Sodium 2 was reinserted into the Na2 site and remained stable, coordinated by backbone carbonyl oxygens and the sulfur atom of the M311 side chain.

We extracted closely spaced snapshots from before and from after rotation of the T308 side chain to investigate, if partial dehydration and rotation of the T308 side chain improved coordination of sodium at the Na2 site. We re-inserted sodium into its original position in the Na2 site and carried out 50 ns long equilibrium MD simulations. The protein was stable in all simulations: we observed Cα RMSD values between 0.12 and 0.16 nm for the TM helices. The low RMSD showed that the pulling rate of the SMD simulation was slow enough to allow the transporter to proceed along the low energy path without significant force induced distortions. Sodium bound to the Na2 site was unstable before rotation of the T308 side chain and dissociated from the Na2 binding site in simulations using starting conformations extracted before rotation of T308 (Fig 4A). In contrast, sodium was stable after rotation. The structural adjustments, which were only possible after breaking of the hydrogen bond between T308 and P304, needed a few nanoseconds to propagate through TM7A to form the Na2 sodium binding site, because sodium was found to remain stably bound to the position in the crystal structure (of Tl^+^) only 6 ns after rotation of T308. We observed that backbone carbonyl oxygen atoms of the last turn of helix TM7A improved coordination of the sodium ion. The fully assembled Na2 binding site was formed by the carbonyl oxygen atoms at the C-terminal ends of helix TM7A, the first helix of the HP2 loop and by the sulfur atom of the M311 side chain. Na2 remained stably coordinated in the inserted position in the simulations started from the last two tested time points (See Fig 4B and 4C), indicative of a completed Na2 sodium binding site. We also found (using APBS [40]) a considerable increase in the negative potential at the Na2 site.

A slightly different binding site for sodium 2 had been proposed from 2 ns equilibrium simulations, followed by free energy calculations [22]. T308 was found in the rotated state after 2 ns [22] and sodium 2 had moved from the crystallographically observed Tl^+^ position and interacted with the side chain of T308. Thereby, sodium had moved away from the center of the region with negative electrostatic potential created by the helix dipoles of helix TM7A and the first helix of the HP2 loop. We carried out three independent simulations to investigate the possibility that a rotated T308 in the start structure could stabilize the sodium in the Na2 binding site. Sodium shifted to the same position with direct contact to the hydroxyl group of the T308 side chain as proposed earlier [22] in the beginning of each simulation. This position resulted metastable. In two simulations sodium dissociated from this intermediate site after 5 ns and diffused into the water phase. In the third simulation, sodium detached within the first nanosecond, suggesting that this could be a intermediate state on the path of sodium binding to the Na2 site.

### Ionic interactions prevent unproductive transport events

**Fig 5:**
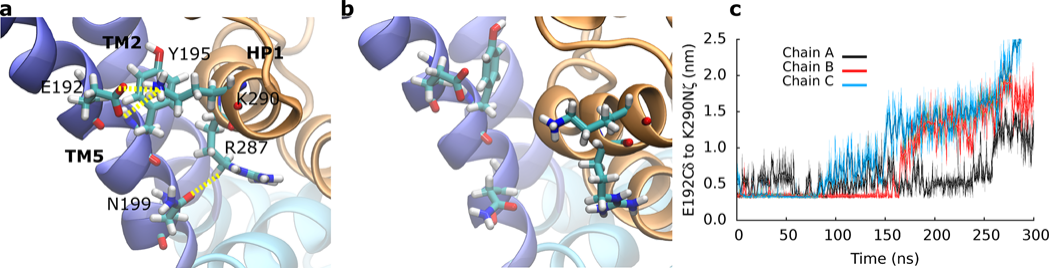
Interactions at the cytosolic site. Interaction network at the cytosolic side (a) in the outward-facing and (b) the intermediate state. (c) Time evolution of the salt bride between E192 and K290. The transporter moves only after opening of the salt bridge.

The outward-facing transporter is stabilized at the cytosolic side by an interaction network between the trimerization domain and the transport domain. The network includes residues E192, Y195 and N199 on helix TM5 (trimerization domain) and residues R287 and K290 on the HP1 loop (transport domain) (Fig 5A and 5B). We observed salt bridges between residues E192 and K290 and between residues N199 and R287. The salt bridge between E192 and K290 was further stabilized by a cation-π interaction of K290 with the aromatic ring of Y195. The distance of the salt bridge between atoms Cδ of E192 and atom Nζ of K290 increased from direct residue contact in the outward-facing conformation to a broad distribution around 1.5 nm in the intermediate state (Fig 5C). Opening of the salt bridge (E192-K290) was accompanied by rotation of Y195. The distance increased further, when the inward facing state was reached. The second salt bridge between residues N199 and R287 showed similar behavior.

### Experimental validation of the Na2 sodium-binding site

**Fig 6:**
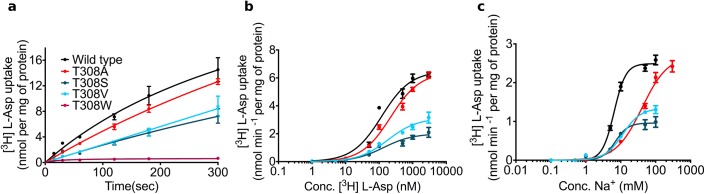
Effects of T308 mutations on substrate uptake. (a) Uptake of [^3^H] L-aspartate into GltPh containing proteoliposomes. We could not observe aspartate uptake for the T308W mutation. (b) [^3^H] L-aspartate uptake into proteoliposomes as a function of [^3^H] L-aspartate concentration and (c) [^3^H] L-aspartate uptake into proteoliposomes as a function of sodium concentration. The data are from three independent experiments; error bars show s.e.m.

Our simulations suggested that (i) the Na2 sodium-binding site forms during substrate translocation and that (ii) the side chain of residue T308 is a key player of its maturation. We challenged this hypothesis by experimentally measuring the involvement of T308 in binding and transport. We created three types of mutations: (i) the T308W mutation introduced an amino acid that would be too large to allow for transport; (ii) the smaller T308A and the T308V mutations were designed to remove the hydroxyl group; (iii) the T308S mutation retained the hydroxyl group (Fig 6 and Table 1). The T308W mutation abolished transport as predicted (Fig 6A). The T308A and T308V mutations showed increased Michaelis-Menten constant (K_m_) values for the substrate L-Asp and for the co-transported sodium ions (Fig 6B and 6C). The transport stoichiometry of GltPh predicts that three sodium ions are co-transported with substrate. A Hill coefficient of ~2.5 for sodium indicates strong cooperativity [20]. This was recapitulated in our experiments: we observed for wild type GltPh a Hill coefficient of 2.5 ± 0.3 for sodium-supported substrate uptake (Fig 6C). The cooperativity was reduced in both mutants, which removed the hydroxyl group, i.e. to 1.1 ± 0.1 and 1.7 ± 0.3 in T308A and T308V, respectively. The drop in cooperativity is consistent with the prediction that the mutations affected binding of sodium to the Na2 site. The K_m_ of substrate uptake was increased from 119 ± 18 nM in wild type GltPh to 183 ± 26 nM and 220 ± 24 nM for T308A and T308V, respectively. The T308S mutation maintained the hydroxyl group at the γ position. We observed an almost 3 fold decrease in the maximal transport rate (V_max_), but only a very small change in the Hill coefficient (2.3 ± 0.7). The K_m_ for sodium (9.2 ± 2.3 mM) and substrate (134 ± 27) were unchanged.

**Table 1:** Transport of Na^+^ and L-Asp in GltPh.

Mutant	K_m_ for Na^+^ (mM)	Hill coefficient for Na^+^ uptake	K_m_ for Asp (nM)
Wild type^a^	8.4 ± 1.3	2.5 ± 0.3	119 ± 18
T308A	47.6 ± 6.3	1.1 ± 0.1	220 ± 24
T308S	9.2 ± 2.3	2.3 ± 0.7	134 ± 27
T308V	18.4 ± 6.3	1.7 ± 0.3	183 ± 26
T308W	N.D.	N.D.	N.D.

^a^Errors are represented as s.e.m.

**Fig 7:**
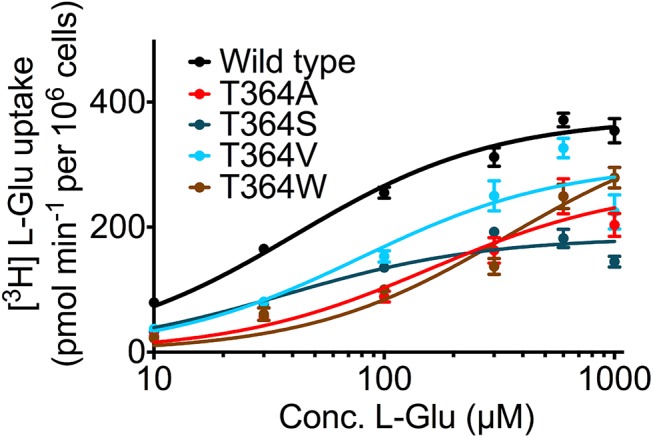
Substrate uptake by human EAAT3. Uptake of [^3^H] L-glutamate into HEK293 cells expressing the human EAAT3 transporter that carrying the same mutations (A, S, V, and W) at the corresponding residue T364. The data are from three independent experiments; error bars show s.e.m.

GltPh is predicted to share its fold with the human EAATs. Sequence alignments showed that T308 and P304 are conserved between the archeal and the human transporters. We therefore reasoned that mutations in human EAATs would recapitulate the effects seen in GltPh, if the structure-function relationship was shared. The same mutations (tryptophan, alanine, valine and serine) of corresponding residue T364 were introduced to the human EAAT3. Experiments were carried out in transiently transfected HEK293 cells (Fig 7). Mutations of EAAT3 reproduced the findings seen in GltPh: the K_m_ for transport was not changed in EAAT3-T364S (37 ± 7 μM) as compared to wild type EAAT3 (41 ± 4 μM), but increased in EAAT3-T364V (79 ± 20 μM), in EAAT-T364A (159 ± 48 μM) and EAAT3-T364W (341 ± 82 μM). In contrast to GltPh, we found residual transport in EAAT3-T364W, indicating that EAAT3 might be more tolerant.

## Discussion

During substrate translocation, accessibility of the substrate binding site changed from the extracellular to the intracellular side. We used SMD simulations to investigate the structural details of this large scale conformational change. Analysis of the conformational landscape revealed the existence of an intermediate state, which is similar to the conformation observed in the crystal structure (PDB ID: 3V8G) [13], showing an RMSD of 0.3–0.4 nm. Binding of substrate and sodium has been shown to induce the closure of the HP2 gate [25,26,36]. We found that the Na2 sodium-binding site is not yet fully formed in the outward-occluded state [12]. The sodium ion escaped in spite of weak restraints, which were imposed to keep it in place. The Na2 site was found to mature during the transition to the intermediate state. The side chain of residue T308 played a pivotal role: breaking of the P304-T308 hydrogen bond weakened the structural stability of the last turn of the TM7A helix (Fig 3). The backbone carbonyl oxygen atoms could therefore adjust and optimally coordinate the sodium ion in the Na2 site. The structural adaptation increased the negative electrostatic potential at the Na2 site, which became more attractive for the positively charged sodium ion. Sodium binding can neutralize the negative potential, thereby removing an energy barrier for the transition to the intermediate state. Therefore, we carried out additional MD simulations to confirm that the Na2 site matured during the transition to the intermediate state. Simulations starting from closely spaced snapshots showed that the Na2 site became competent for sodium binding only after breaking of the hydrogen bond between the side chain of T308 and the backbone carbonyl oxygen of P304. Our results indicate that breaking of this hydrogen bond is the key event that triggers the structural adjustments that lead to maturation of the Na2 site. This observation is in line with the predicted sequence of binding events in GltPh: sodium 3, followed by sodium 1, substrate and sodium 2 [22,25,26]. Electrophysiological measurements determined that binding to the outward-facing EAAT3 transporter occurred in at least two steps: (i) fast binding of substrate and sodium ion(s) followed by (ii) slow binding of one additional sodium ion [41]. The transporter translocated substrate only after the slow sodium binding event.

**Fig 8:**
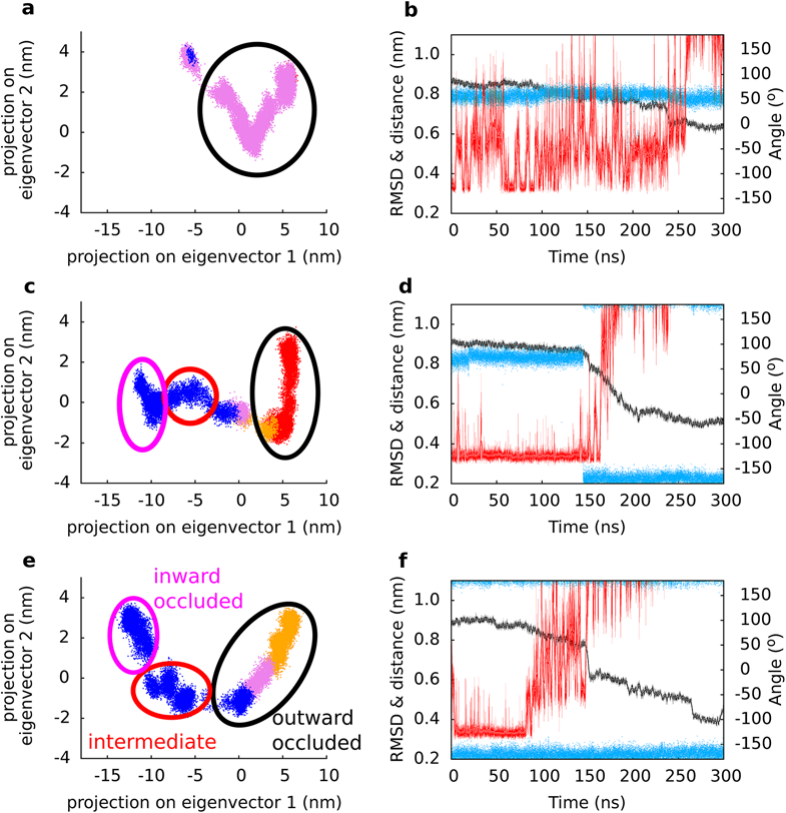
Correlation analysis. Panel (a, c, e) show movements of a GltPh protomer along the two largest eigenvectors (translation and rotation of the domains relative to each other) observed in the simulations of protomer A, B and C, respectively. Each dot represents the projection of one frame in the trajectory onto the 2 dimension of eigenvectors 1 (translation) and 2 (rotation). Four distinct periods of the trajectory are color coded: The first part of the trajectory (red) represents the outward-occluded state. A shift to orange marked the rotation of T308. Rotation of side chain of Y195 notifies the transition to the third period (pink). The fourth region shown in blue represents the period after opening of the intracellular interaction network. The intermediate state is reached in the populated region in the middle of the plot, after opening of the intracellular interaction network. Panel (b, d, f) show an overlay of the time evolution of the RMSD (black), the rotational state of the side chain of T308 (blue) and the side chain–side chain distance between E192 and K290 (red) of protomer A, B and C, respectively.

We used SMD simulations to study substrate uptake. A drop in the RMSD of each chain reflects a movement towards the inward-occluded state. Analyses of the trajectories revealed two crucial local events that accompanied the transition through the intermediate state: (i) formation of the Na2 site (Fig 3) and (ii) opening of the interaction network at the intracellular side (Fig 5). We applied correlation analyses independently to each protomer (Fig 8) to determine, if these local changes occurred first and thereby triggered the global changes (See S8 Fig for analyses of all 3 simulations). The two largest global motions consisted of the translation of the transport domain *vs*. the trimerization domain (principal component 1), and a rotation of the transport *vs*. the trimerization domain (principal component 2). Only protomer B and C reached the inward-occluded state. The time points of local changes were color-code mapped onto the 2d projection. The analyses revealed that (i) formation of the Na2 site occurred before the transition to the intermediate state. (ii) Opening of the interaction network was also required prior to formation of the intermediate state. It followed after maturation of the Na2 site. Mutations of residues contributing to the intracellular interaction network (specifically K290A) were shown to reduce substrate transport [42], most likely by destabilizing the outward-facing state. We found the conformation of the Y195 side chain to be a sensor for early onset of conformational changes at the inner site of the transporter. The long side chains of E192 and K290 allowed for limited structural adjustment before rupture. Only once the salt bridge opened could substrate translocation proceed. The same results were obtained in the analyses of the other two simulations (S7 Fig). Our correlation analyses therefore suggest that the local changes are *the key switches*, which prepare GltPh for the global changes and should therefore control the transition through the transport cycle.

These observations have important implications for the transport mechanism. They predict that the local events represent essential shifts in the energy hypersurface along the transport cycle: (i) binding of substrate and sodium to the Na1 and Na3 site stabilizes the outer gate HP2 loop in the closed state [36]. (ii) Occupation of the Na2 site reduces the energy barrier for the transition to the intermediate state. (iii) Opening of the interaction network at the cytosolic site allows transport and trimerization domain to move relative to each other. Accessibility of the substrate binding site changes from extracellular to intracellular. (iv) Opening of the intracellular gate allows for substrate release and ion dissociation [28]. These events indicate that the role of sodium binding is to modify the energy hypersurface of the transporter and, as a consequence, to allow for directional transport. Directionality can be maintained as long as the internal sodium concentration remains low to reset the transporter [43].

We confirmed this mechanism by performing radiotracer flux experiments of wild type and mutant GltPh. Residues P304 and T308 in TM7A are conserved from archaea to humans, preserving the hydrogen bonding property. The conservative T308S mutation, which maintains the hydroxyl group, had little effect on Hill coefficient and K_m_. Removal of the hydroxyl group (mutated to alanine and valine) was predicted to alter the K_m_ and the Hill coefficient. We observed a larger effect for the T308A mutant as compared to the T308V mutant. Valine and threonine are both β-branched amino acids and have helix destabilizing properties, while alanine does not have the same property, suggesting that an imperfectly stabilized TM7A helix is important for transporter function. These experimental results support the transport mechanism model developed from our simulations.

Our results revealed mechanistic insights into the mechanics of substrate translocation, which are conserved between GltPh and human EAATs. Controlled glutamate uptake by the EAATs is essential for brain function: a reduced uptake is associated with excess glutamate in the synaptic cleft that can lead to excitotoxicity and neurodegenerative diseases including amyotrophic lateral sclerosis, stroke, epilepsy or Huntington’s disease. Currently no drug on the market can enhance EAAT transporter function. It should be of interest to identify compounds, which could provide pharmacological leads that apply such a new strategy for patient treatment. The interactions and structural changes, which we identified, represent checkpoints to ensure correct binding of substrate and sodium ions before the conformational transition. We speculate that the Na2 site fulfills three functions: (i) it allows the transporter to reach an intermediate state only if substrate and three sodium ions are bound. (ii) It prevents the escape of bound substrate to the extracellular environment. (iii) Futile cycling events of sodium ions bound transporter are precluded in the absence of substrate, because closure of the HP2 loop and the presence of substrate and sodium in the Na2 site are needed. This arrangement is an elegant solution to harness the energy stored in the sodium gradient and to prevent unproductive cycling, which only translocates sodium ions. Accumulation of internal sodium would be harmful to neurons and glia cells, because the electrochemical gradient across the cell membrane is eventually dissipated by futile cycling.

## Material and Methods

### System preparation and equilibrium simulations

Simulations were carried out using the GROMACS 4.5.4 package [44]. The OPLS [45] all atom force field was applied to GltPh, the POPC membrane was described using the Berger lipids [46] force field. The inward-facing (PDB-ID: 3KBC) [12] and the outward-occluded crystal structure of GltPh (PDB-ID: 2NWX) [11] were selected; missing residues (side chains and residues) were added using MODELLER version 9.8 [47]. The third sodium ion was placed into the previously reported ion binding site [23]. GltPh was embedded in a pre-equilibrated POPC bilayer using the g_membed [48] procedure and solvated with water, electro-neutralized and a physiological concentration of NaCl (150 mM). The SPC water model [49] was used. Lennard-Jones interactions were calculated until a cutoff of 1.0 nm. Long range electrostatic interactions were treated using PME [50]. Bond lengths were constrained using LINCS [51]. The temperature was maintained at 310 K applying the v-rescale thermostat [52]; pressure was set to 1 bar and controlled by the Berendsen barostat [53], coupling protein, membrane, and water/ions separately. The assembled system was equilibrated by first relaxing membrane and solvent. The protein was then gradually relaxed by reducing the restraints on the protein atoms in five steps [54] (each 2.5 ns simulation) applying a restraining force on the protein of 1000, 100, 10, 1, 0.1 kJ mol^-1^ nm^-2^. Production runs were then started. We carried out equilibrium simulations starting from two independently prepared inward and the outward-occluded state, and nine simulations starting from snapshots extracted from one SMD simulation.

### Steered molecular dynamics

We used SMD simulations [55] to induce a conformational change from the outward- to the inward-facing conformation. We selected the same number of Cα atoms from both the trimerisation and the transport domain. The spring force constant was set to 1000.0 kJ mol^-1^ nm^-2^, applied only along the membrane normal (Z-direction). Pilot simulations with different force constants indicated that higher force constants would lead to a force profile that showed an increasing level of fluctuations and a larger number of force peaks in the force profile (See S1 Fig), indicative for entanglement and forced barrier-crossing events. These force peaks seemed to depend on the applied sprint constant, because they gradually disappeared with decreasing restraining force. Repeated simulations showed that the peaks did not always appear at the same time points. Correct behavior of the protein could therefore only be expected with a force constant of 1000.0 kJ mol^-1^ nm^-2^. All simulations were repeated three times. Pilot simulations were also performed to identify the optimal pulling velocity. We selected a pulling rate of 0.00001 nm ps^-1^. This velocity is slow enough to give GltPh time to relax all not coupled degrees of freedom and fast enough to be still accessible to MD simulations. Higher pulling speed resulted in unphysical structural deformations and increasingly larger peaks in the force time contour. We carried out three SMD simulations starting from independently prepared systems.

### Plasmids and site-directed mutagenesis

Eric Gouaux (Oregon Health & Science University) generously donated wild type GltPh. Site-directed mutagenesis was performed using Quick Change Lightning site directed mutagenesis kit (Agilent Technologies). All mutations were confirmed using DNA sequencing.

### Protein purification and reconstitution

GltPh was purified as described previously [56]. Briefly, membranes were isolated, solubilized with (1%) n-dodecyl-β-D-maltopyranoside (DDM) (Anatrace Detergents), and the protein was purified using Ni-NTA resin. The detergent was then exchanged to (0.15%) n-decyl-β-D-maltopyranoside (DM) (Anatrace Detergents). The protein was reconstituted into liposomes using the method described previously with slight modifications [57]. Escherichia coli total lipids (Avanti Polar lipids Inc.) and 1-palmitoyl-2-oleoyl-sn-glycero-3-phosphocholine (Avanti Polar Lipids Inc.) were mixed at a ratio of 3:1, dried under nitrogen and re-suspended in internal buffer (100 mM KCl, 20 mM HEPES, pH 7.5). The lipid mixture was briefly sonicated using a point tip sonicator. The lipid suspension was flash frozen in liquid nitrogen and thawed at room temperature multiple times. Liposomes were formed by extrusion through 400 nm polycarbonate membranes (Avanti Polar Lipids Inc.) and destabilized with Triton X-100 prior to addition of protein at a ratio of 0.25 μg protein per mg lipid. The protein-lipid mixture was kept at room temperature for 30 minutes. Detergent was then removed using SM2 biobeads (Biorad). The protein-lipid mixture was incubated and gently agitated with three consecutive batches of biobeads (20 mg ml^-1^). The biobeads were subsequently removed by filtration. The resulting proteoliposomes were concentrated by centrifugation at 150,000 g for 30 min in a Beckman centrifuge, re-suspended at 100 mg lipid mL^-1^ and either used immediately or flash frozen in liquid nitrogen and stored at −80°C.

### Proteoliposome transport assay

Transport of [^3^H] L-aspartate by wild type and mutant GltPh was assayed by applying the protocol modified from Gaillard et al., 1996 [56]. Briefly, proteoliposomes were loaded with buffer A (100 mM KCl, 20 mM HEPES, pH 7.5) by repeated freeze and thaw cycles followed by extrusion. The uptake was initiated by diluting the proteoliposomes into buffer B (100 mM NaCl, 20 mM HEPES, pH 7.5, 1 μM valinomycin and the indicated concentrations of [^3^H] L-aspartate) pre-warmed to 30°C. At each time point, a 200 μL aliquot was removed and diluted 10 fold into ice cold stop buffer C (100 mM LiCl, 20 mM HEPES, pH 7.5), followed by filtration over nitrocellulose filters (0.22 μm pore size, Merck Millipore). The filters were washed three times with 2 mL of ice-cold quench buffer and assayed for radioactivity. Sodium dependence of [^3^H] L-aspartate transport was measured by varying extraliposomal Na^+^ concentration from 0.1 to 300 mM. Osmolarity was balanced using choline chloride (Sigma Aldrich). [^3^H] L-aspartate concentrations used for determining the sodium dependence were 100 nM for wild type and around K_m_ for other mutants. Background levels of uptake were measured by diluting proteoliposomes into buffer A containing 1 μM valinomycin and the indicated concentrations of [^3^H] L-aspartate.

### Transport assay of EAAT3 in HEK293 cells

The plasmid PL28 encoding for the human excitatory amino acid transporter 3 (EAAT3) was a kind gift by Peter Larsson (Oregon Health & Science University). The EAAT3 cDNA was cloned into pmGFP-C1. The point mutations T364A, T364V, T364S, and T364W were generated using Quick Change Lightning site-directed mutagenesis kit II (Agilent technologies) and confirmed by DNA sequencing. Human embryonic kidney 293 (HEK293) cells were cultured in Dulbecco’s Modified Eagle’s Medium (DMEM), which was supplemented with 10% fetal bovine serum at 37°C and 5% CO_2_. Cells were transiently transfected with wild type mGFP-EAAT3 with the EAAT3 mutants T364A, T364V, T364S, and T364W using the calcium phosphate method. Cells were seeded (0.1*10^5^ cells per well) on 48-well plates pre-coated with poly-D-lysine. The transport assay was performed 48 hours after transfection. Cell were washing with 500 μl of Krebs HEPES buffer (10 mM HEPES, 130 mM NaCl, 1.3 mM KH_2_PO_4_, 1.5 mM CaCl_2_, 0.5 mM MgSO_4_, pH adjusted to 7.4 using NaOH). Uptake was initiated by incubating cells with increasing concentrations (10, 30, 100, 300, 600 and 1000 μM) of unlabeled substrate L-glutamate (Sigma Aldrich) containing tracer amount (100 nM) of radiolabeled [^3^H] L-glutamate (Perkin Elmer). The uptake was terminated after 10 min by washing with ice cold Krebs HEPES buffer. Cells were lysed with 500 μl of 1% sodium-dodecyl-sulfate and radioactivity was measured with the Liquid Scintillation Analyzer. Non-specific uptake was determined by pre-incubation with the inhibitor L-trans-pyrrolidine-2,4-dicarboxylate (PDC) (Sigma Aldrich) 10 min prior and during incubation (100μM) and subtracted from the total counts.

## Supporting Information

S1 Fig Distance vs. time and force vs. time plot. Panel (a) shows the change in distance over time with respect to the starting conformation of the 2NWX crystal structure, panel (b) shows the time evolution of the force acting on the two domains. The reference point (or reference distance) is changed at a constant velocity in the SMD simulations. The applied force is calculated from the difference between the reference distance and the instantaneous distance using Hook’s law.
(TIFF)

S2 Fig Selection of the spring constant strength. Pilot simulations were performed, using a pull rate of 0.0001 nm/ps and applying four different spring force constants: 1000, 5000, 10000 and 15000 kJ mol^-1^ nm^-2^. Force profiles are shown until the first GltPh protomer passed the inward-occluded conformation. The shape of the force profiles indicated that force constants higher than 1000 kJ mol^-1^ nm^-2^ do not allow for sufficient side chains disentanglement, bearing the danger of observing erratic behavior induced by strong coupling.
(TIF)

S3 Fig Structural stability of trimerisation and transport domains. Panels a, b, and c show the time evolution of the distance between S278 (HP1) and G354 (HP2) on the tip of the respective loops. A larger distance was observed in chain A in all the three simulations (a, b, c). Overall structural stability of the trimerisation (d, e, f) and transport domain (g, h, i) was estimated by measuring the RMSD to the starting structure. The RMSD of individual domains remained stable for major part of the SMD simulation.
(TIFF)

S4 Fig Comparison of the inward-occluded conformation as observed in the simulation with the crystal structure (3KBC). Conformation of GltPh extracted from the simulation (also shown in Fig 1E) is shown in panel a and rotated in panel c. This conformation which is closest (by RMSD) to the inward facing crystal structure is depicted in panel b and d. Residues K55C and A364C form a disulfide bond in the crystal. The two residues are in close proximity in our simulations of wild type GltPh.
(TIF)

S5 Fig Salt bridge between residue E192 and K290. Panel (a, b, c): Time evolution of the salt-bridge distance was measured between atoms E192-Cδ and K290-Nζ of three independent simulations. The salt bridge is formed between the residues from the trimerisation and transport domain. Time evolution of dihedral angle χ2 of Y195 is shown in panel (d, e, f).
(TIFF)

S6 Fig Formation of Na2 binding site. Panel (a, b, c): Time evolution of the T308 side chain dihedral angle χ1 is shown for three independent simulations. Panel (d, e, f): The level of hydration of T308 was estimated as the number of water molecules within 0.6 nm of T308-Cα atom.
(TIFF)

S7 Fig RMSD to the intermediate state. The RMSD plot measures the structural deviation of each chain for three independent SMD simulations to the crystallographically observed intermediate state (PDB ID: 3V8G). Chain A is shown in black, chain B in red, chain C in blue.
(TIFF)

S8 Fig Correlation analysis. Principal component analysis of three independent simulations (panels (a, b, c): chain A, B, C of simulation 1; panels (d, e, f): chain A, B, C of simulation 2; panels (g, h, i): chain A, B, C of simulation 3); the figures are as depicted in the main manuscript (Fig 8). Briefly, movement of a GltPh protomer along the two largest eigenvectors (translation, rotation of the domains relative to each other) observed in the simulations. Each dot represents the projection of one frame in the trajectory onto the 2 dimensions of eigenvectors 1 and 2. Four distinct periods of the trajectory are color coded: The first part of the trajectory (red) represents the outward-occluded state before rotation of the T308 side chain. A shift to orange marked the rotation of T308. Rotation of the side chain of Y195 marks the transition to the third period (pink). The fourth region shown in blue represents the period after opening of the intracellular interaction network. The intermediate state is reached in the populated region in the middle of the plot, after opening of the intracellular interaction network.
(TIFF)

## References

[1] Danbolt NC. Glutamate uptake. Prog Neurobiol. 2001;65: 1–105. 11369436 10.1016/s0301-0082(00)00067-8

[2] Zerangue N, Kavanaugh MP. Flux Coupling in Neuronal Glutamate Transporter. Nature. 1996;383: 634–637. 8857541 10.1038/383634a0

[3] Levy LM, Warr O, Attwell D. Stoichiometry of the Glial Glutamate Transporter GLT-1 Expressed Inducibly in a Chinese Hamster Ovary Cell Line Selected for Low Endogenous Na+ -Dependent Glutamate Uptake. J Neurosci. 1998;18: 9620–9628. 9822723 10.1523/JNEUROSCI.18-23-09620.1998 PMC6793325

[4] Owe SG, Marcaggi P, Attwell D. The ionic stoichiometry of the GLAST glutamate transporter in salamander retinal glia. J Physiol. 2006;577: 591–9. 10.1113/jphysiol.2006.116830 17008380 PMC1890427

[5] Foran E, Trotti D. Glutamate Transporters and the Excitotoxic Path to Motor Neuron Degeneration in Amyotrophic Lateral Sclerosis. Antioxidants Redox Signal. 2009;11: 1587–1602. 10.1089/ars.2009.2444 PMC2842587 19413484

[6] Maragakis NJ, Rothstein JD. Glutamate transporters: animal models to neurologic disease. Neurobiol Dis. 2004;15: 461–73. 10.1016/j.nbd.2003.12.007 15056453

[7] Yi J-H, Hazell AS. Excitotoxic mechanisms and the role of astrocytic glutamate transporters in traumatic brain injury. Neurochem Int. 2006;48: 394–403. 10.1016/j.neuint.2005.12.001 16473439

[8] Sheldon AL, Robinson MB. The role of glutamate transporters in neurodegenerative diseases and potential opportunities for intervention. Neurochem Int. 2007;51: 333–55. 10.1016/j.neuint.2007.03.012 17517448 PMC2075474

[9] Kim K, Lee S-G, Kegelman TP, Su Z-Z, Das SK, Dash R, et al Role of excitatory amino acid transporter-2 (EAAT2) and glutamate in neurodegeneration: opportunities for developing novel therapeutics. J Cell Physiol. 2011;226: 2484–93. 10.1002/jcp.22609 21792905 PMC3130100

[10] Yernool D, Boudker O, Jin Y, Gouaux E. Structure of a glutamate transporter homologue from Pyrococcus horikoshii. Nature. 2004;431: 811–8. 10.1038/nature03018 15483603

[11] Boudker O, Ryan RM, Yernool D, Shimamoto K, Gouaux E. Coupling substrate and ion binding to extracellular gate of a sodium-dependent aspartate transporter. Nature. 2007;445: 387–93. 10.1038/nature05455 17230192

[12] Reyes N, Ginter C, Boudker O. Transport mechanism of a bacterial homologue of glutamate transporters. Nature. 2009;462: 880–5. 10.1038/nature08616 19924125 PMC2934767

[13] Verdon G, Boudker O. Crystal structure of an asymmetric trimer of a bacterial glutamate transporter homolog. Nat Struct Mol Biol. 2012;19: 355–7. 10.1038/nsmb.2233 22343718 PMC3633560

[14] Bendahan A, Armon A, Madani N, Kavanaugh MP, Kanner BI. Arginine 447 plays a pivotal role in substrate interactions in a neuronal glutamate transporter. J Biol Chem. 2000;275: 37436–42. 10.1074/jbc.M006536200 10978338

[15] Rosental N, Bendahan A, Kanner BI. Multiple consequences of mutating two conserved beta-bridge forming residues in the translocation cycle of a neuronal glutamate transporter. J Biol Chem. 2006;281: 27905–15. 10.1074/jbc.M600331200 16870620

[16] Teichman S, Kanner BI. Aspartate-444 is essential for productive substrate interactions in a neuronal glutamate transporter. J Gen Physiol. 2007;129: 527–39. 10.1085/jgp.200609707 17535962 PMC2151622

[17] Teichman S, Qu S, Kanner BI. The equivalent of a thallium binding residue from an archeal homolog controls cation interactions in brain glutamate transporters. Proc Natl Acad Sci U S A. 2009;106: 14297–302. 10.1073/pnas.0904625106 19706515 PMC2732801

[18] Teichman S, Qu S, Kanner BI. Conserved asparagine residue located in binding pocket controls cation selectivity and substrate interactions in neuronal glutamate transporter. J Biol Chem. 2012;287: 17198–205. 10.1074/jbc.M112.355040 22493292 PMC3366809

[19] Akyuz N, Georgieva ER, Zhou Z, Stolzenberg S, Cuendet MA, Khelashvili G, et al Transport domain unlocking sets the uptake rate of an aspartate transporter. Nature. 2015;518: 68–73. 10.1038/nature14158 25652997 PMC4351760

[20] Ryan RM, Compton ELR, Mindell J a. Functional characterization of a Na+-dependent aspartate transporter from Pyrococcus horikoshii. J Biol Chem. 2009;284: 17540–8. 10.1074/jbc.M109.005926 19380583 PMC2719393

[21] Groeneveld M, Slotboom D-J. Na(+):aspartate coupling stoichiometry in the glutamate transporter homologue Glt(Ph). Biochemistry. 2010;49: 3511–3. 10.1021/bi100430s 20349989

[22] Heinzelmann G, Baştuğ T, Kuyucak S. Free energy simulations of ligand binding to the aspartate transporter Glt(Ph). Biophys J. 2011;101: 2380–8. 10.1016/j.bpj.2011.10.010 22098736 PMC3218339

[23] Huang Z, Tajkhorshid E. Identification of the third Na+ site and the sequence of extracellular binding events in the glutamate transporter. Biophys J. 2010;99: 1416–25. 10.1016/j.bpj.2010.06.052 20816053 PMC2931724

[24] Larsson HP, Wang X, Lev B, Baconguis I, Caplan D a, Vyleta NP, et al Evidence for a third sodium-binding site in glutamate transporters suggests an ion/substrate coupling model. Proc Natl Acad Sci U S A. 2010;107: 13912–7. 10.1073/pnas.1006289107 20634426 PMC2922246

[25] Huang Z, Tajkhorshid E. Dynamics of the extracellular gate and ion-substrate coupling in the glutamate transporter. Biophys J. 2008;95: 2292–300. 10.1529/biophysj.108.133421 18515371 PMC2517027

[26] Shrivastava IH, Jiang J, Amara SG, Bahar I. Time-resolved mechanism of extracellular gate opening and substrate binding in a glutamate transporter. J Biol Chem. 2008;283: 28680–90. 10.1074/jbc.M800889200 18678877 PMC2568915

[27] DeChancie J, Shrivastava IH, Bahar I. The mechanism of substrate release by the aspartate transporter GltPh: insights from simulations. Mol Biosyst. 2011;7: 832–42. 10.1039/c0mb00175a 21161089 PMC3227142

[28] Zomot E, Bahar I. Intracellular gating in an inward-facing state of aspartate transporter Glt(Ph) is regulated by the movements of the helical hairpin HP2. J Biol Chem. 2013;288: 8231–7. 10.1074/jbc.M112.438432 23386619 PMC3605641

[29] Holley DC, Kavanaugh MP. Interactions of alkali cations with glutamate transporters. Philos Trans R Soc Lond B Biol Sci. 2009;364: 155–61. 10.1098/rstb.2008.0246 18977733 PMC2674104

[30] Tao Z, Rosental N, Kanner BI, Gameiro A, Mwaura J, Grewer C. Mechanism of cation binding to the glutamate transporter EAAC1 probed with mutation of the conserved amino acid residue Thr101. J Biol Chem. 2010;285: 17725–33. 10.1074/jbc.M110.121798 20378543 PMC2878536

[31] Bastug T, Heinzelmann G, Kuyucak S, Salim M, Vandenberg RJ, Ryan RM. Position of the third Na+ site in the aspartate transporter GltPh and the human glutamate transporter, EAAT1. PLoS One. 2012;7: e33058 10.1371/journal.pone.0033058 22427946 PMC3302783

[32] Gu Y, Shrivastava IH, Amara SG, Bahar I. Molecular simulations elucidate the substrate translocation pathway in a glutamate transporter. Proc Natl Acad Sci U S A. 2009;106: 2589–94. 10.1073/pnas.0812299106 19202063 PMC2637273

[33] Grazioso G, Limongelli V, Branduardi D, Novellino E, De Micheli C, Cavalli A, et al Investigating the mechanism of substrate uptake and release in the glutamate transporter homologue Glt(Ph) through metadynamics simulations. J Am Chem Soc. 2012;134: 453–63. 10.1021/ja208485w 22092197

[34] Stolzenberg S, Khelashvili G, Weinstein H. Structural Intermediates in a Model of the Substrate Translocation Path of the Bacterial Glutamate Transporter Homologue GltPh. J Phys Chem B. 2012;116: 5372–5383. 10.1021/jp301726s 22494242 PMC3350225

[35] Erkens GB, Hänelt I, Goudsmits JMH, Slotboom DJ, van Oijen AM. Unsynchronised subunit motion in single trimeric sodium-coupled aspartate transporters. Nature. 2013;502: 119–23. 10.1038/nature12538 24091978

[36] Focke PJ, Moenne-Loccoz P, Larsson HP. Opposite movement of the external gate of a glutamate transporter homolog upon binding cotransported sodium compared with substrate. J Neurosci. 2011;31: 6255–62. 10.1523/JNEUROSCI.6096-10.2011 21508248 PMC3096012

[37] Heinzelmann G, Bastug T, Kuyucak S. Mechanism and energetics of ligand release in the aspartate transporter GltPh. J Phys Chem B. 2013;117: 5486–96. 10.1021/jp4010423 23590433

[38] Jardetzky O. Simple Allosteric Model for Membrane Pumps. Nature. 1966;211: 969–70. 5968307 10.1038/211969a0

[39] Rosental N, Kanner BI. A conserved methionine residue controls the substrate selectivity of a neuronal glutamate transporter. J Biol Chem. 2010;285: 21241–8. 10.1074/jbc.M109.087163 20424168 PMC2898430

[40] Baker NA, Sept D, Joseph S, Holst MJ, Mccammon JA. Electrostatics of nanosystems: Application to microtubules and the ribosome. Proc Natl Acad Sci U S A. 2001;98: 10037–10041. 10.1073/pnas.181342398 11517324 PMC56910

[41] Watzke N, Bamberg E, Grewer C. Early Intermediates in the Transport Cycle of the Neuronal Excitatory Amino Acid Carrier EAAC1. J Gen Physiol. 2001;117: 547–62. 11382805 10.1085/jgp.117.6.547 PMC2232401

[42] Akyuz N, Altman RB, Blanchard SC, Boudker O. Transport dynamics in a glutamate transporter homologue. Nature. Nature Publishing Group; 2013;502: 114–8. 10.1038/nature12265 23792560 PMC3829612

[43] Grewer C, Gameiro A, Zhang Z, Tao Z, Braams S, Rauen T. Glutamate forward and reverse transport: From molecular mechanism to transporter-mediated release after ischemia. IUBMB Life. 2009;60: 609–619. 10.1002/iub.98.Glutamate PMC2632779 18543277

[44] Hess B, Kutzner C, van der Spoel D, Lindahl E. GROMACS 4: Algorithms for Highly Efficient, Load-Balanced, and Scalable Molecular Simulation. J Chem Theory Comput. American Chemical Society; 2008;4: 435–447. 10.1021/ct700301q 26620784

[45] Jorgensen WL, Maxwell DS, Tirado-Rives J. Development and Testing of the OPLS All-Atom Force Field on Conformational Energetics and Properties of Organic Liquids. J Am Chem Soc. American Chemical Society; 1996;118: 11225–11236. 10.1021/ja9621760

[46] Berger O, Edholm O, Jähnig F. Molecular dynamics simulations of a fluid bilayer of dipalmitoylphosphatidylcholine at full hydration, constant pressure, and constant temperature. Biophys J. 1997;72: 2002–13. 10.1016/S0006-3495(97)78845-3 9129804 PMC1184396

[47] Šali A, Blundell TL. Comparative Protein Modelling by Satisfaction of Spatial Restraints. J Mol Biol. 1993;234: 779–815. 10.1006/jmbi.1993.1626 8254673

[48] Wolf MG, Hoefling M, Aponte-santamaría C, Grubmüller H, Groenhof G. g _ membed: Efficient Insertion of a Membrane Protein into an Equilibrated Lipid Bilayer with Minimal Perturbation. J Comput Chem. 2010;31: 2169–2174. 10.1002/jcc 20336801

[49] Berendsen HJC, Postma JPM, van Gunsteren WF, Hermans J. Interaction Models for Water in Relation to Protein Hydration. Intermolecular Forces: The Jerusalem Symposia on Quantum Chemistry and Biochemistry. 1981. pp. 331–42.

[50] Darden T, York D, Pedersen L. Particle mesh Ewald: An N⋅log(N) method for Ewald sums in large systems. J Chem Phys. 1993;98: 10089–92.

[51] Hess B, Bekker H, Berendsen HJC, Fraaije JGEM. LINCS: A linear constraint solver for molecular simulations. J Comput Chem. 1997;18: 1463–1472.

[52] Bussi G, Donadio D, Parrinello M. Canonical sampling through velocity rescaling. J Chem Phys. 2007;126: 014101 17212484 10.1063/1.2408420

[53] Berendsen HJC, Postma JPM, van Gunsteren WF, DiNola A, Haak JR. Molecular dynamics with coupling to an external bath. J Chem Phys. 1984;81: 3684–90.

[54] Stockner T, Montgomery TR, Kudlacek O, Weissensteiner R, Ecker GF, Freissmuth M, et al Mutational analysis of the high-affinity zinc binding site validates a refined human dopamine transporter homology model. PLoS Comput Biol. 2013;9: e1002909 10.1371/journal.pcbi.1002909 23436987 PMC3578762

[55] Isralewitz B, Gao M, Schulten K. Steered molecular dynamics and mechanical functions of proteins. Curr Opin Struct Biol. 2001;11: 224–230. 10.1016/S0959-440X(00)00194-9 11297932

[56] Gaillard I, Slotboom D, Knol J, Lolkema JS, Konings WN. Purification and Reconstitution of the Glutamate Carrier GltT of the Thermophilic Bacterium Bacillus stearothermophilus. Biochemistry. 1996;2960: 6150–6156. 10.1021/bi953005v 8634258

[57] Geertsma ER, Nik Mahmood N a B, Schuurman-Wolters GK, Poolman B. Membrane reconstitution of ABC transporters and assays of translocator function. Nat Protoc. 2008;3: 256–66. 10.1038/nprot.2007.519 18274528

