# Supplementary figures and images for: Refinement of the Central Steps of Substrate Transport by the Aspartate Transporter GltPh: Elucidating the Role of the Na2 Sodium Binding Site
Source: PLoS Comput Biol. 2015 Oct 20;11(10):e1004551. doi: 10.1371/journal.pcbi.1004551 (PMC4618328; doi:10.1371/journal.pcbi.1004551)

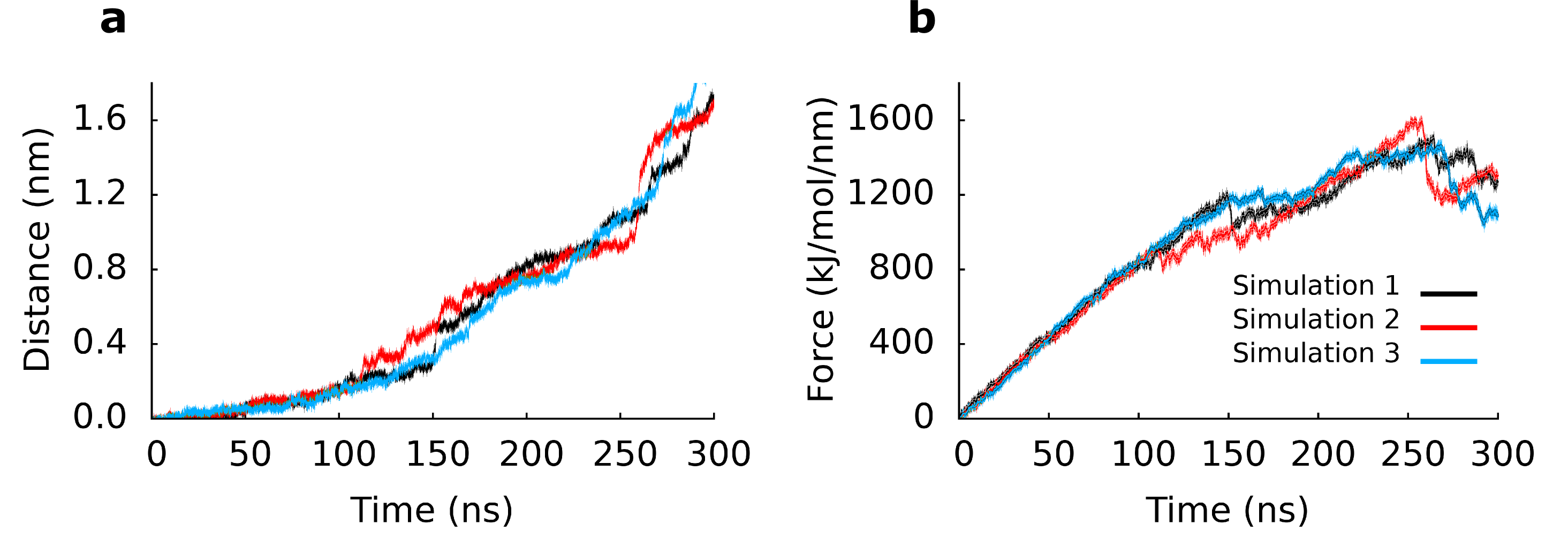

Supplement: S1 Fig — Panel (a) shows the change in distance over time with respect to the starting conformation of the 2NWX crystal structure, panel (b) shows the time evolution of the force acting on the two domains. The reference point (or reference distance) is changed at a constant velocity in the SMD simulations. The applied force is calculated from the difference between the reference distance and the instantaneous distance using Hook’s law. (TIFF) [file pcbi.1004551.s001.tiff]

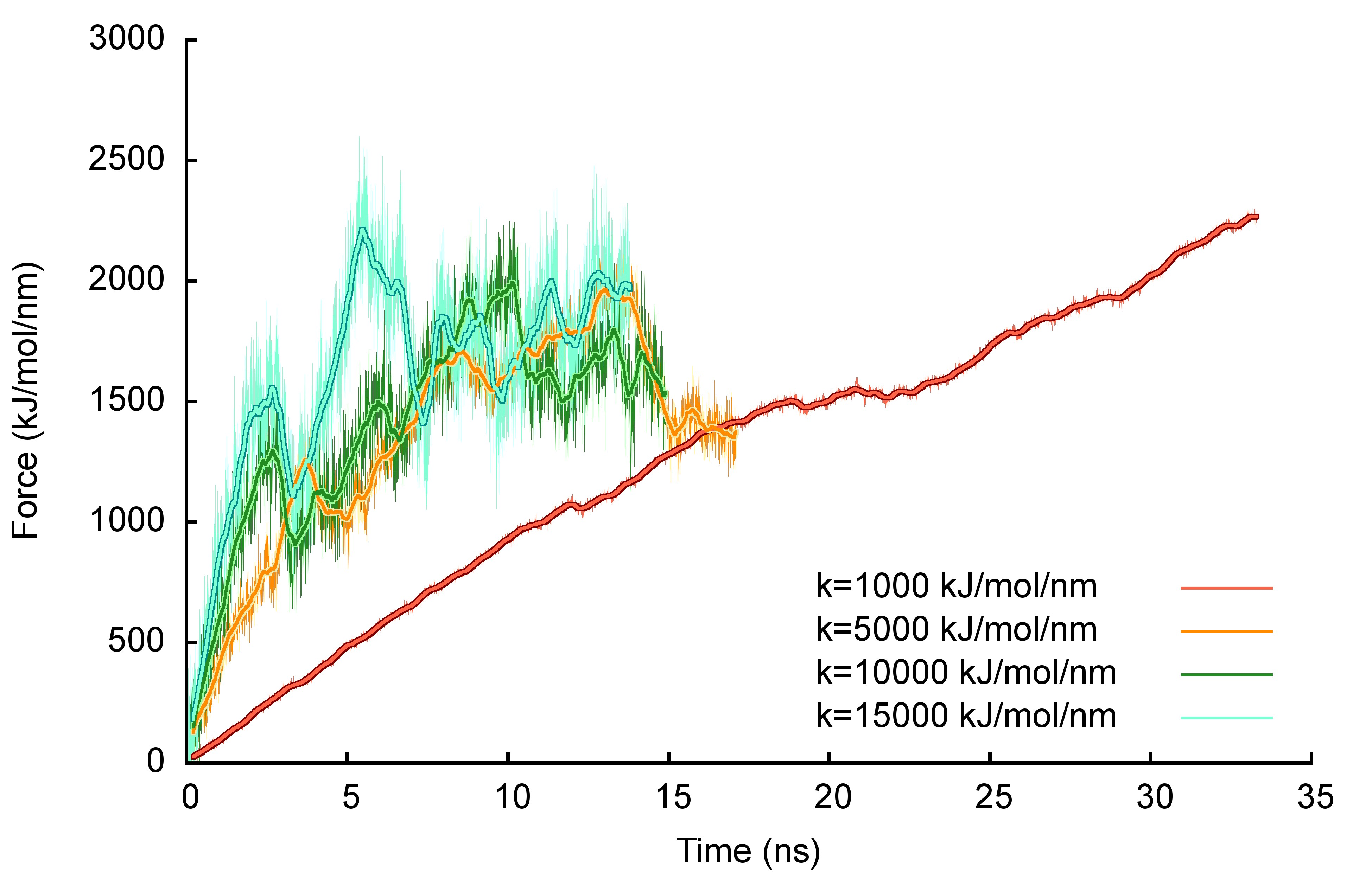

Supplement: S2 Fig — Pilot simulations were performed, using a pull rate of 0.0001 nm/ps and applying four different spring force constants: 1000, 5000, 10000 and 15000 kJ mol-1 nm-2. Force profiles are shown until the first GltPh protomer passed the inward-occluded conformation. The shape of the force profiles indicated that force constants higher than 1000 kJ mol-1 nm-2 do not allow for sufficient side chains disentanglement, bearing the danger of observing erratic behavior induced by strong coupling. (TIF) [file pcbi.1004551.s002.tif]

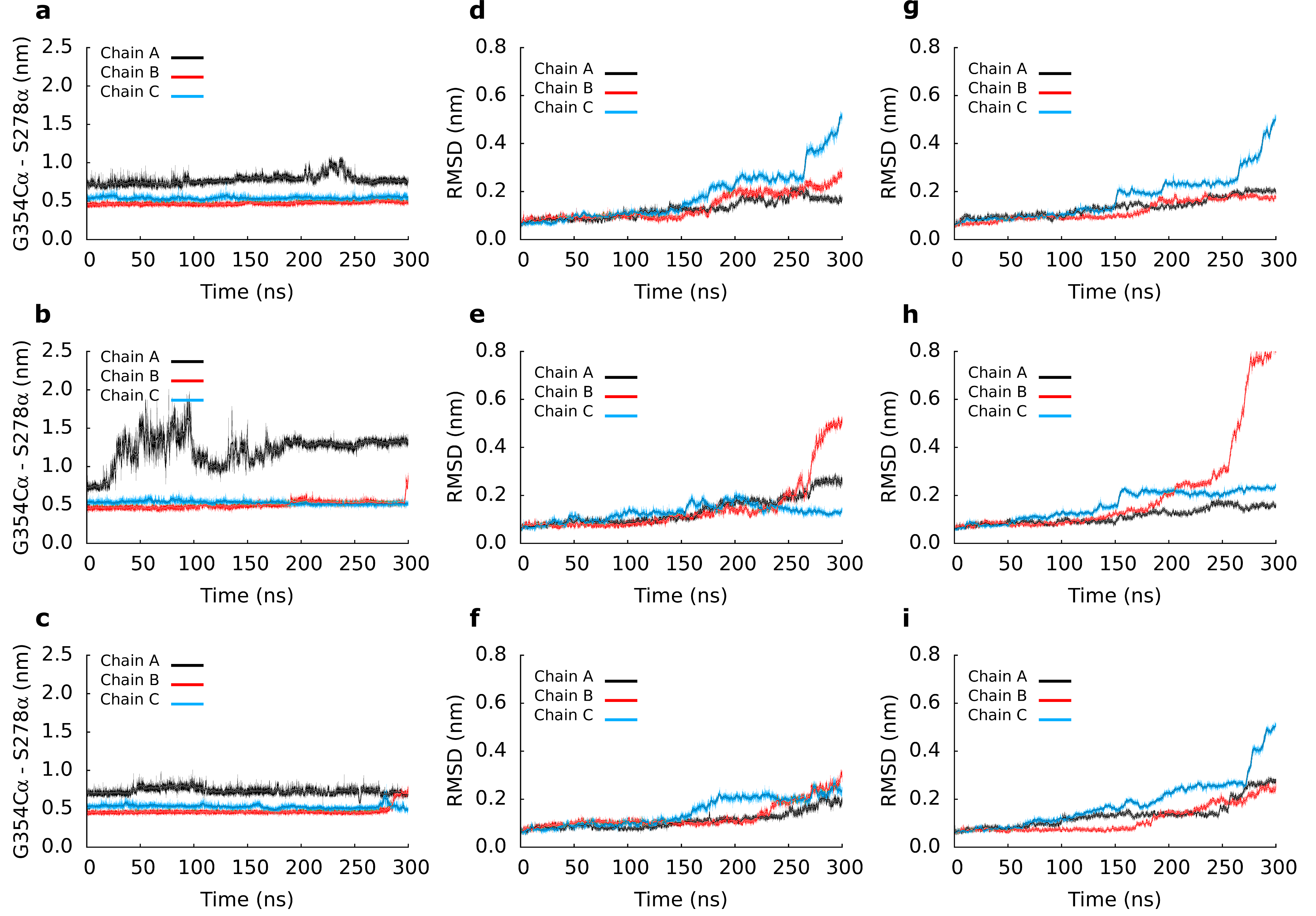

Supplement: S3 Fig — Panels a, b, and c show the time evolution of the distance between S278 (HP1) and G354 (HP2) on the tip of the respective loops. A larger distance was observed in chain A in all the three simulations (a, b, c). Overall structural stability of the trimerisation (d, e, f) and transport domain (g, h, i) was estimated by measuring the RMSD to the starting structure. The RMSD of individual domains remained stable for major part of the SMD simulation. (TIFF) [file pcbi.1004551.s003.tiff]

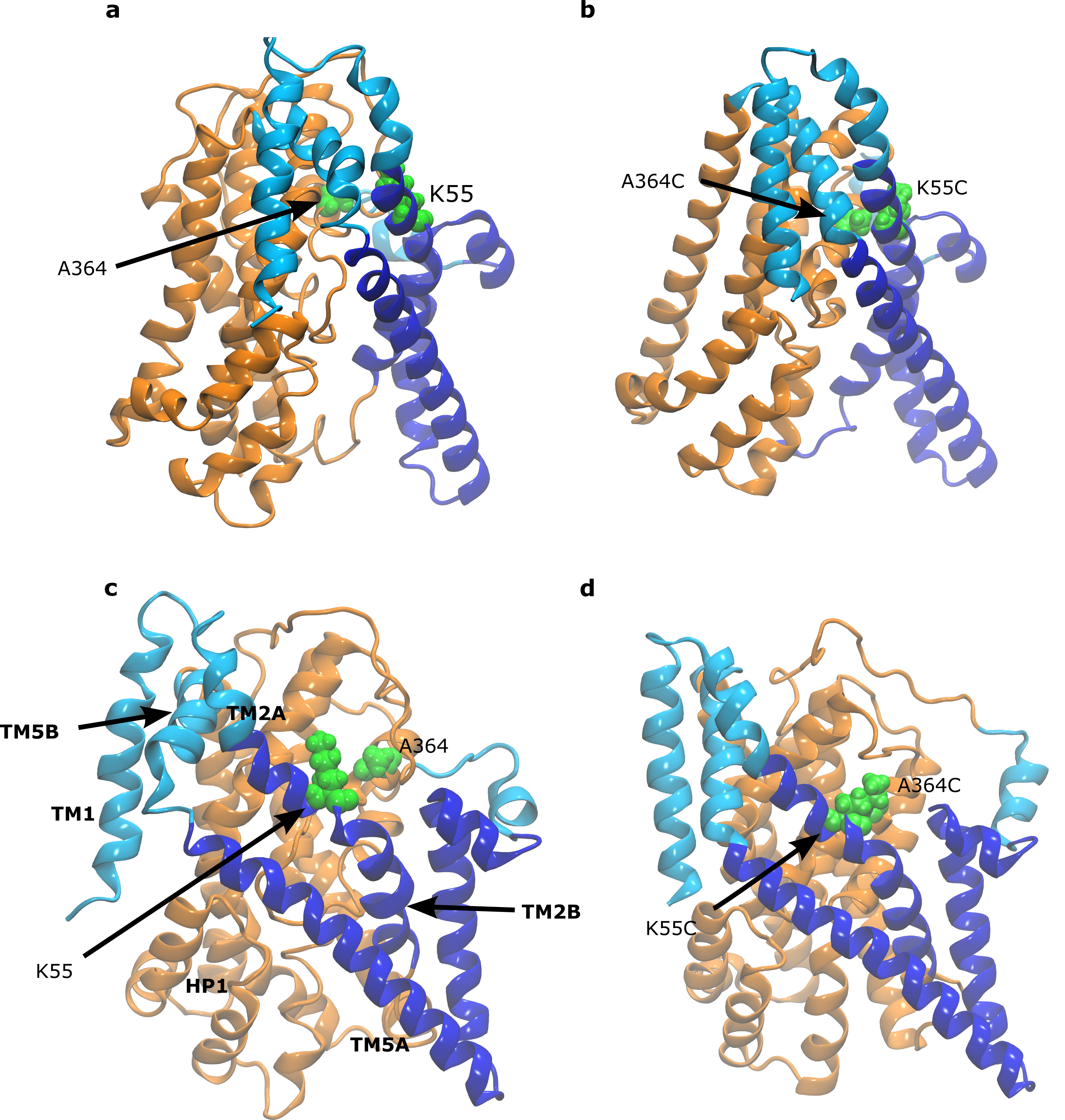

Supplement: S4 Fig — Conformation of GltPh extracted from the simulation (also shown in Fig 1E) is shown in panel a and rotated in panel c. This conformation which is closest (by RMSD) to the inward facing crystal structure is depicted in panel b and d. Residues K55C and A364C form a disulfide bond in the crystal. The two residues are in close proximity in our simulations of wild type GltPh. (TIF) [file pcbi.1004551.s004.tif]

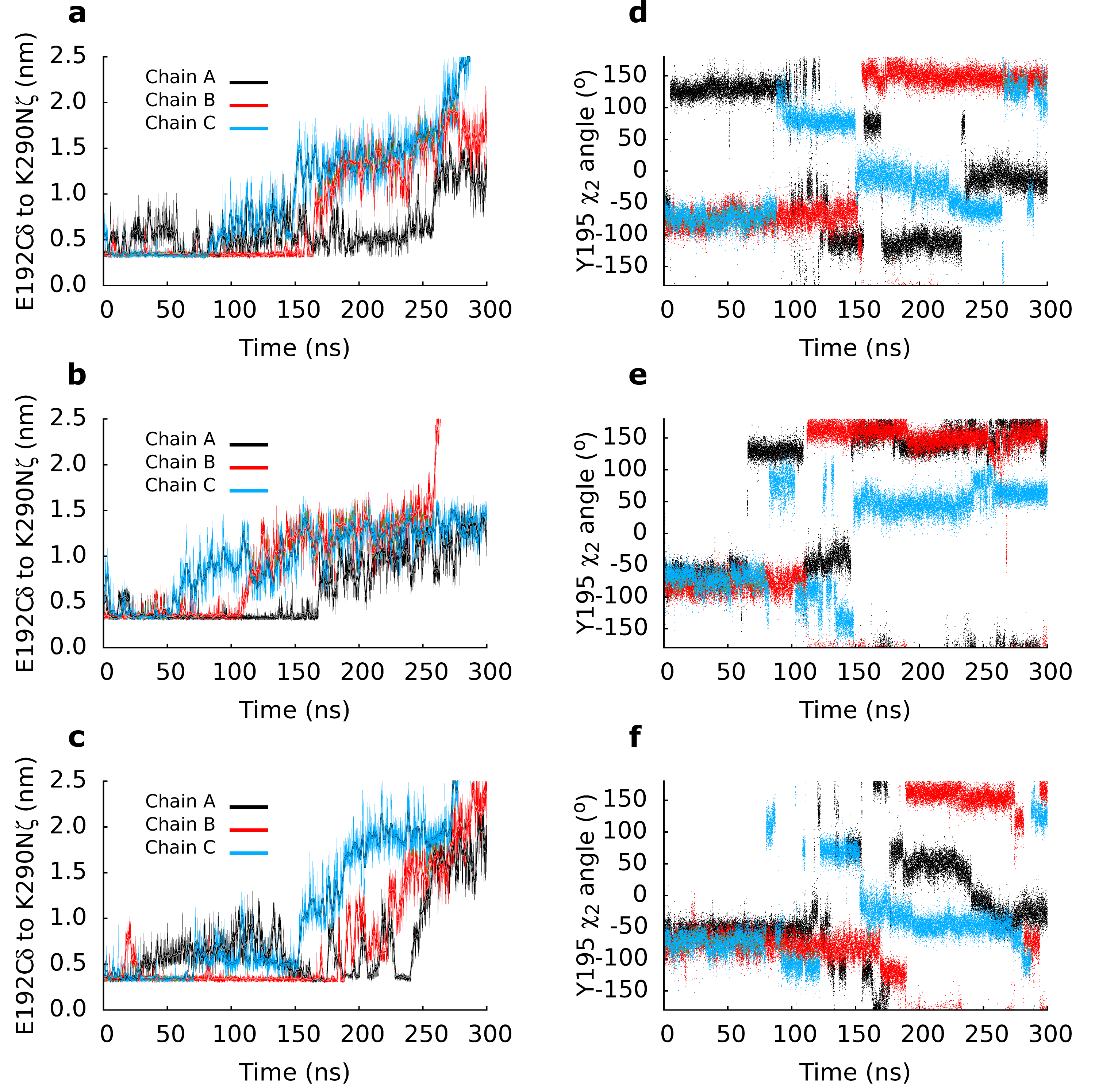

Supplement: S5 Fig — Panel (a, b, c): Time evolution of the salt-bridge distance was measured between atoms E192-Cδ and K290-Nζ of three independent simulations. The salt bridge is formed between the residues from the trimerisation and transport domain. Time evolution of dihedral angle χ2 of Y195 is shown in panel (d, e, f). (TIFF) [file pcbi.1004551.s005.tiff]

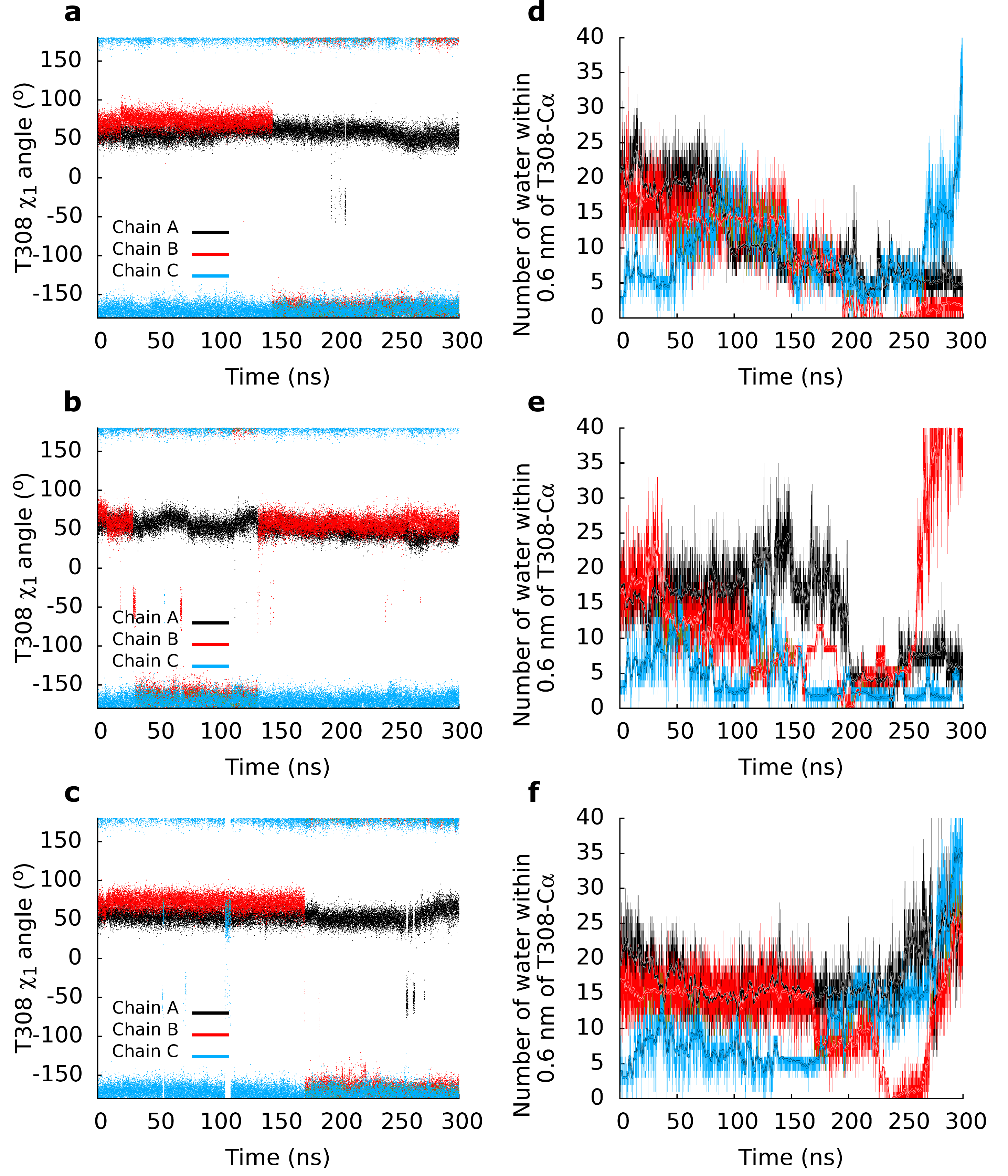

Supplement: S6 Fig — Panel (a, b, c): Time evolution of the T308 side chain dihedral angle χ1 is shown for three independent simulations. Panel (d, e, f): The level of hydration of T308 was estimated as the number of water molecules within 0.6 nm of T308-Cα atom. (TIFF) [file pcbi.1004551.s006.tiff]

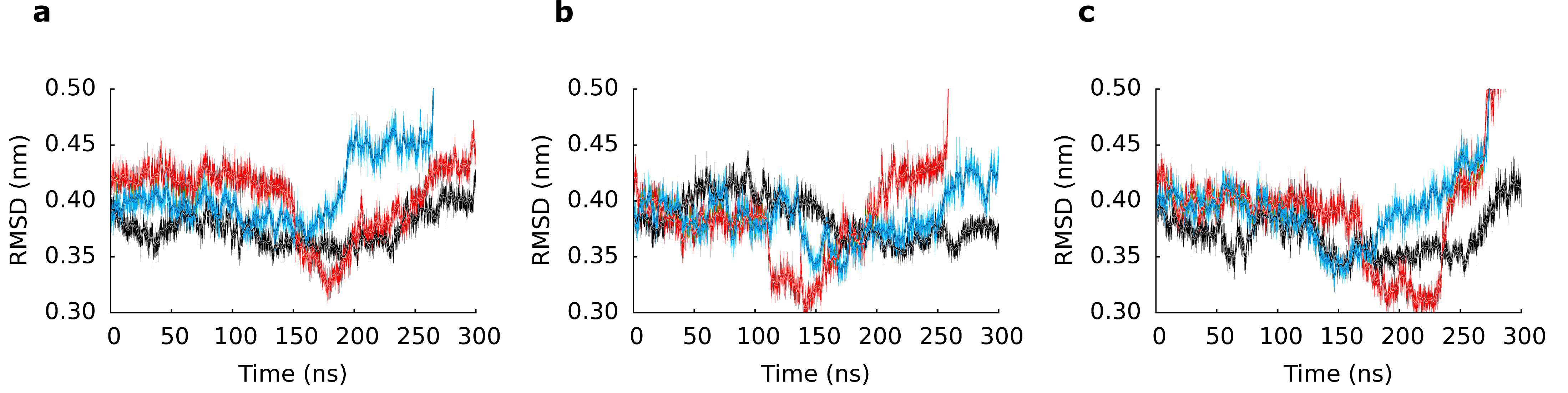

Supplement: S7 Fig — The RMSD plot measures the structural deviation of each chain for three independent SMD simulations to the crystallographically observed intermediate state (PDB ID: 3V8G). Chain A is shown in black, chain B in red, chain C in blue. (TIFF) [file pcbi.1004551.s007.tiff]

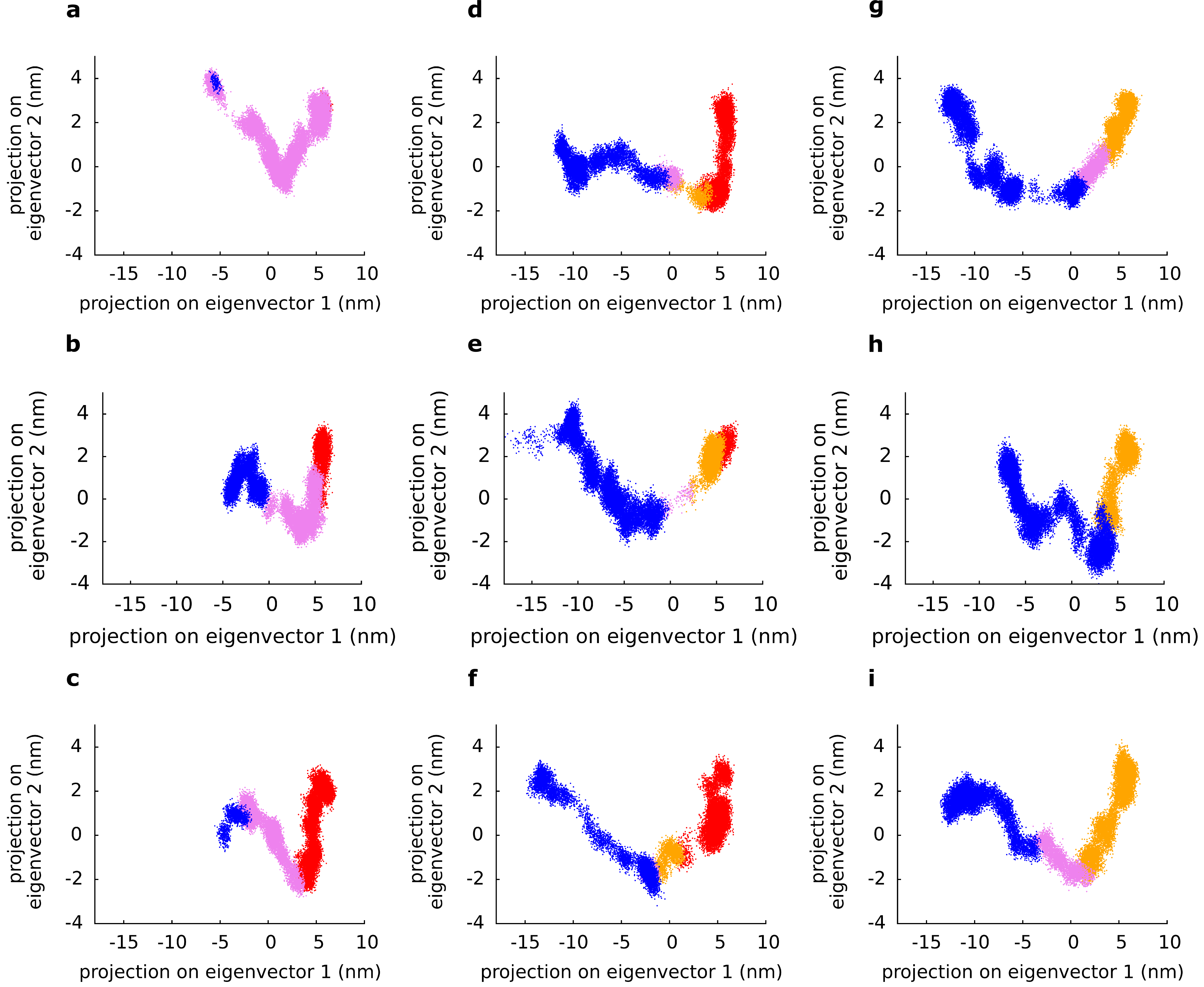

Supplement: S8 Fig — Principal component analysis of three independent simulations (panels (a, b, c): chain A, B, C of simulation 1; panels (d, e, f): chain A, B, C of simulation 2; panels (g, h, i): chain A, B, C of simulation 3); the figures are as depicted in the main manuscript (Fig 8). Briefly, movement of a GltPh protomer along the two largest eigenvectors (translation, rotation of the domains relative to each other) observed in the simulations. Each dot represents the projection of one frame in the trajectory onto the 2 dimensions of eigenvectors 1 and 2. Four distinct periods of the trajectory are color coded: The first part of the trajectory (red) represents the outward-occluded state before rotation of the T308 side chain. A shift to orange marked the rotation of T308. Rotation of the side chain of Y195 marks the transition to the third period (pink). The fourth region shown in blue represents the period after opening of the intracellular interaction network. The intermediate state is reached in the populated region in the middle of the plot, after opening of the intracellular interaction network. (TIFF) [file pcbi.1004551.s008.tiff]
